# Recovery of oculomotor nerve palsy after endovascular management of posterior communicating artery aneurysms

**DOI:** 10.4102/sajr.v24i1.1887

**Published:** 2020-08-31

**Authors:** Elkharbash Abdurahman, Khatija Amod, Duncan Royston, Rohen Harrichandparsad

**Affiliations:** 1 Department of Radiology, University of KwaZulu-Natal, Durban, South Africa; 2 Department of Radiology, Inkosi Albert Luthuli Central Hospital, University of KwaZulu-Natal, Durban, South Africa; 3 Lake, Smit & Partners, Durban, South Africa; 4 Department of Neurosurgery, Inkosi Albert Luthuli Central Hospital, University of KwaZulu-Natal, Durban, South Africa

**Keywords:** ONP, PcomA, clipping versus coiling, diagnostic radiology, Endovascular coiling, Oculomotor nerve recovery

## Abstract

**Background:**

Oculomotor nerve palsy (ONP) is a common clinical presentation of posterior communicating artery (PcomA) aneurysms. It remains unclear if patients have a better rate of recovery after surgical clipping or endovascular coiling.

**Objectives:**

The main objectives of this study were to assess the overall rate of ONP recovery after endovascular coiling of PcomA aneurysms, as well as to determine the associated predictive factors of oculomotor nerve recovery.

**Method:**

We retrospectively evaluated the demographic, clinical, and radiological characteristics and the outcome of consecutive patients presenting with PcomA aneurysms treated by endovascular coiling from January 2012 to November 2016 with at least 1 year clinical and radiological follow-up. Statistical analysis was applied to determine the association between ONP recovery and the demographic, clinical and radiological variables.

**Results:**

A total of 91 patients with PcomA aneurysms were treated endovascularly. Thirty-four patients (22 women and 12 men) with ONP related to PcomA aneurysms were included. The mean age of the patients was 49.8 years. Subarachnoid haemorrhage was present in 27 patients. The mean aneurysm size was 6.7 mm. The overall rate of recovery was 88.2%. Complete nerve recovery was seen in 16 (47%) patients and partial recovery was observed in 14 (41.2%) patients, whilst 4 (11.8%) patients remained unchanged after treatment. The non-posterolateral direction of the aneurysm showed a tendency towards better recovery compared to the posterolateral projection (*p* = 0.06).

**Conclusion:**

Endovascular coiling of PcomA aneurysms in patients with ONP resulted in a cure or improvement of oculomotor nerve dysfunction in the majority of patients.

## Introduction

Posterior communicating artery (PcomA) aneurysms are one of the most common aneurysms encountered by neurosurgeons and neuro-interventional radiologists, representing 50% of all internal carotid artery (ICA) aneurysms. They are the second most common aneurysms overall (25% of all aneurysms).^1^ Oculomotor nerve palsy (ONP) is a common clinical presentation of both ruptured and unruptured PcomA aneurysms and has been reported to vary from 35% to 45%.^2,3^ However, the exact pathophysiologic mechanisms underlying aneurysmal third nerve palsy, remain unknown. There are many theories published about the pathophysiologic mechanism of ONP, including direct mechanical compression of the third nerve by enlargement of the aneurysmal sac in the suprasellar cistern, nerve injury by arterial pulsation of the aneurysm, as well as by pressure from arterial bleeding because of rupture of the aneurysm, and irritation from subarachnoid haemorrhage.^4,5^

The treatment of ONP-related to PcomA aneurysms is still controversial and has been limited by the small series of cases. The first surgical series of aneurysmal third nerve palsy was published in 1947 by Jefferson et al.,^6^ who recommended carotid ligation, principally to prevent recurrent haemorrhage. The later introduction of the operating microscope and the development of microneurosurgical techniques, resulted in clipping being considered the best option for the management of PcomA aneurysms.^7,8^ With advances in technology, endovascular therapy has been used more frequently, as efficiently and less invasively.^9,10^ However, it remains unclear if patients would have a better rate of recovery after surgical clipping or endovascular coiling.^11,12,13,14^ Controversies exist regarding the predictive factors that might contribute to ONP recovery after management, as there are many conflicting reports from previous studies.

The primary aim of this study was to assess the rate of ONP recovery related to PcomA aneurysms in an institution where coiling is the first policy. The secondary aim was to determine the predictive factors of oculomotor nerve recovery.

## Material and methods

### Study population

This was a retrospective, descriptive and analytic study of patients who presented with oculomotor nerve palsies related to PcomA aneurysms and underwent endovascular treatment with coiling from January 2012 to November 2016 in the Neurosurgery Department at Inkosi Albert Luthuli Central Hospital (IALCH) in South Africa. Patients with at least 1 year clinical and radiological follow-up were included in the study.

### Data collection

Patient data were obtained from the computer database of the Hospital Information System at IALCH. Demographics, clinical presentation and ophthalmologic examination were recorded from the patients’ medical files. Images were accessed via the Radiological Information System and Picture Archiving and Communication System. Data on the endovascular procedure and the course of ophthalmologic and angiographic follow-up were obtained.

### Pre-operative and postoperative evaluation

Complete ONP was defined as patients with all of the following: diplopia, ptosis, ophthalmoplegia and pupillary dysfunction. Partial (incomplete) ONP was defined as patients with one or two of the above. The following factors were assessed: demographic data (gender and age), clinical data (co-morbidities, degree of ONP at presentation, aneurysm rupture status before treatment and length of ONP before treatment) and radiological data (size and aneurysm direction). Postoperative evaluation of ONP was classified as unchanged (no improvement), incomplete recovery or complete recovery.

### Statistical analysis

Data are presented as means and ranges for continuous variables and as frequencies for categoric variables. Statistical analyses were performed using the Fisher’s exact test. The level of statistical significance used was *p* < 0.05 for the whole study. Statistical analysis was performed using the Statistical Package for the Social Sciences (SPSS) version 26.0.

### Ethical consideration

Ethical approval to conduct the study was obtained from the Higher Research Committee at the University of KwaZulu-Natal (approval number: BE510/17).

## Results

The study population consisted of 91 patients who each presented with a PcomA aneurysm. Of the 91 patients, 40 (43.9%) had oculomotor palsy and were treated with endovascular coiling. Thirty-four patients were included in this study as they had follow-up data. Of the 34 patients, 30 patients (88.2%) had ONP recovery, whilst 4 patients (11.8%) remained unchanged after endovascular treatment. Of the 30 patients who had ONP recovery, 16 patients had complete recovery of oculomotor nerve function, whilst 14 had an incomplete recovery. The demographic, clinical and radiographic data are presented in Table 1.

**TABLE 1:** Correlation between demographic, clinical and radiographic factors versus recovery of oculomotor nerve palsy after endovascular coiling.

Factors	Variable	Complete recovery (*n* = 16)	Incomplete recovery (*n* = 14)	No improvement (*n* = 4)	*p*
**Demographic**					
**Gender**	**-**	**-**	**-**	**-**	**0.52**
	Male Female	4 12	6 8	2 2	- -
**Age**	**-**	**-**	**-**	**-**	**0.11**
	> 50 years < 50 years	10 6	7 7	0 4	- -
**Clinical**					
**Presence of co-morbidities**	**-**	**-**	**-**	**-**	**0.64**
	Yes No	9 7	6 8	1 3	- -
**ONP at presentation**	**-**	**-**	**-**	**-**	**0.60**
	Complete Partial	14 2	14 0	4 0	- -
**Presence of SAH**	**-**	**-**	**-**	**-**	**0.60**
	Ruptured aneurysm Unruptured aneurysm	13 3	10 4	4 0	- -
**ONP onset to treatment**	**-**	**-**	**-**	**-**	**0.28**
	< 2 weeks > 2 weeks	9 5	5 9	1 3	- -
**Radiographic**					
**Aneurysm size**	**-**	**-**	**-**	**-**	**0.26**
	≤ 6.7 mm > 6.7 mm	11 5	7 7	1 3	- -
**Direction of aneurysm**	**-**	**-**	**-**	**-**	**0.06**
	Posterolateral Non-posterolateral	6 10	9 5	4 0	- -

ONP, oculomotor nerve palsy; SAH, subarachnoid haemorrhage.

### Demographic data in correlation with oculomotor nerve palsy recovery

Of the 34 patients with ONP, 22 (64.7%) were women and 12 (35.3%) were men. The mean age of the patients was 49.8 years (range 14–81 years). No relationship could be found between gender (*p* = 0.52) or age (*p* = 0.11) and ONP recovery.

### Clinical data in correlation with oculomotor nerve palsy recovery

Of the 34 patients who developed ONP, co-morbidities (hypertension/diabetes mellitus) were found in 16 patients (47%), whereas 18 patients (53%) had no comorbidity. At the time of admission, ONP was complete in 32 patients (94.1%) and partial in 2 patients (6.9%). Subarachnoid haemorrhage (SAH), in keeping with aneurysm rupture, was present in 27 patients (79.4%) with ONP, whereas 7 patients (20.6%) had unruptured aneurysms. No statistically significant correlation was found between co-morbidities, the degree of ONP at presentation or SAH versus ONP recovery. Of the 34 patients, the length of ONP before treatment was not documented in two patients. Fifteen patients had early treatment (≤ 14 days), whilst late endovascular coiling (> 15 days) was performed in 17 patients. Early treatment showed a slightly higher tendency towards better ONP recovery compared with late treatment. Nevertheless, the difference did not reach statistical significance (*p* = 0.28).

### Radiological data in correlation with oculomotor nerve palsy recovery

The mean size of the aneurysms was 6.7 mm (range 3 mm – 12.4 mm). Small aneurysms showed a slightly higher tendency towards better ONP recovery compared with larger aneurysms. Nevertheless, the difference did not reach statistical significance (*p* = 0.26). For the 34 patients who developed ONP, aneurysm direction was posterolateral in 19 patients (55.9%), lateral in 10 patients (29.4%), anterolateral in 2 patients (5.9%), inferolateral in 2 patients (5.9%) and medial in 1 patient (27.5%). Patients with a non-posterolateral aneurysm direction indicated a higher tendency towards better ONP recovery compared with the posterolateral group (*p* = 0.06).

## Discussion

The main objectives of this study were to assess the overall rate of ONP recovery related to PcomA aneurysm after endovascular coiling, as well as to determine the associated predictive factors of oculomotor nerve recovery.

In our study, we found that 43.9% of patients with PcomA aneurysms developed ONP. This is comparable to most previous studies, where the range of ONP varied from 34% to 56% amongst patients presenting with PcomA aneurysm.^4,5^ The ONP results because of close anatomical proximity of the PcomA to the third cranial nerve, which may be caused by the pulsing and mass effect of the aneurysm sac, as well as by aneurysm rupture. During the early neurovascular conflict, a conduction block takes place, whilst after longer periods, continuous pulsing or mass effect is likely to affect intraneural circulation, thus leading to nerve ischaemia. If these phenomena become chronic, the occurrence of fibrosis, scarring and necrosis is probable. When SAH is present, there may be several phases of neural damage, from minor axonolysis to direct damage to the nerve when severe bleeding occurs.

**FIGURE 1:**
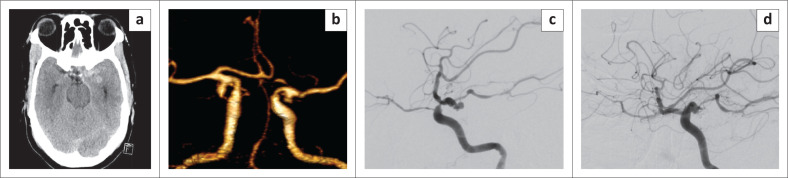
A 65-year old man presented with sudden onset of severe headache. Non-contrast computed tomography brain: (a) showed diffuse subarachnoid haemorrhage (SAH) with a heavier blood load on the left. Computed tomography angiogram (b) showed bilateral common clinical presentation of posterior communicating artery aneurysms. Digital substraction angiography (DSA) of the left internal carotid artery in the lateral projection shows the multilobulated posteriorly directed aneurysm before (c) and after (d) simple coiling embolisation.

**FIGURE 2:**
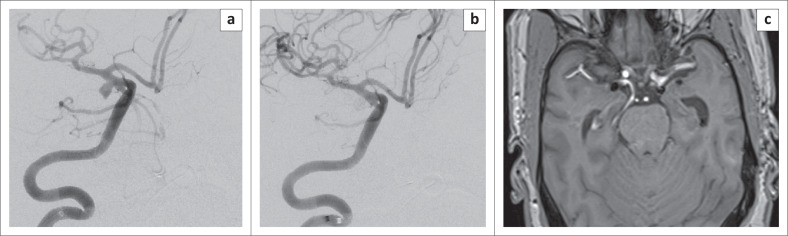
(R) Internal carotid artery digital substraction angiography (DSA) of the same patient 3 months later in the working projection showing the contralateral common clinical presentation of a posterior communicating artery aneurysm: (a) before and (b) after balloon-assisted coiling. Magnetic resonance angiogram at 3 months shows the coil masses bilaterally with no recurrence of the aneurysms.

**FIGURE 3:**
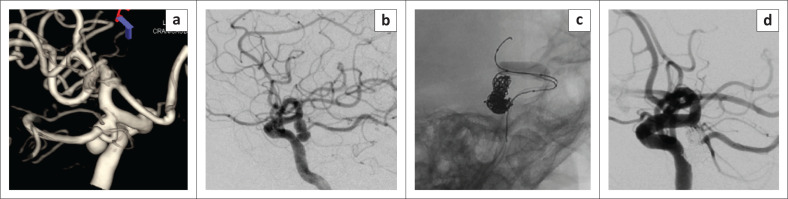
A 64 year old male presents with a complete right third nerve palsy. 3D reconstruction of the (R) ICA DSA shows a PComA aneurysm with the origin of the artery at the neck of the aneurysm (a). Lateral view (b) shows a bilobed PComA aneurysm directed in the typical posterolateral and inferior projection towards the interpeduncular cistern that results in compression of the oculomotor nerve. Unsubtracted view in the working projection (c) shows balloon assisted coiling, using a compliant balloon to protect the origin of the PComA. Control run (d) shows complete occlusion of the aneurysm and normal filling of the PComA branch.

**FIGURE 4:**
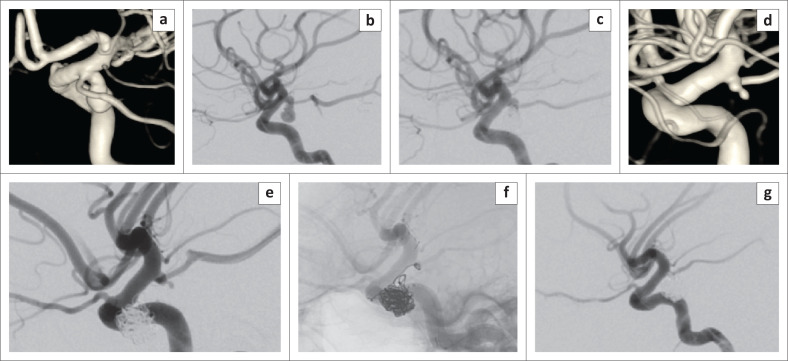
A 27-year-old woman presented with subarachnoid haemorrhage (SAH) and (right) third cranial nerve palsy. Computed tomography angiogram revealed bilateral common clinical presentation of posterior communicating artery aneurysms. The three-dimentional angiogram of the (right) internal carotid artery shows the larger symptomatic aneurysm (a). Lateral view before (b) and after (c) simple coiling. 3D angiogram of the (left) internal carotid artery shows the small, incidental mirror common clinical presentation of posterior communicating artery aneurysm with a relatively wide neck (d). This was treated 3 months later with a stent-assisted coiling (e and f). Control run (g) shows complete occlusion of the aneurysm with normal filling of the internal carotid artery and preservation of the common clinical presentation of the posterior communicating artery.

We assessed the resolution of ONP in a series of 34 patients after endovascular treatment. An overall improvement occurred in 88.2% of the patients. We found that endovascular therapy allowed complete nerve recovery in 47% and partial recovery in 41.2% of patients, whilst only 11.8% remained unchanged. These findings are also in agreement with previous studies that have reported favourable recovery rates with endovascular therapy, ranging from 80% to 90%. In these studies, complete recovery was observed in 40% – 50% of patients, whilst partial recovery was seen in 40% – 45% of patients after endovascular therapy.^15,16^ The exact mechanism by which coiling promotes oculomotor nerve recovery, is not well understood. A commonly proposed explanation is that coiling removes or decreases aneurysmal pulsations, thus enabling nerve recovery.^17^ On the other hand, surgical clipping is likely to cause direct compression or traction, thus offsetting more rapid reduction in pulsatility and mass effect. The recovery rates with surgical clipping have been reported to range between 77% and 87%.^7,8,11,13^ Illustrative cases demonstrating simple coiling, balloon remodelling and stent-assisted coiling techniques are presented in Figures 1–4.

### Correlation of demographic data with oculomotor nerve palsy recovery

The effect of demographics on the final ONP recovery has been shown in several studies. Stiebel-Kalish et al.^18^ and Ahn et al.^19^ reported that older age might decrease the chances of ONP recovery after endovascular coil treatment of PcomA aneurysms. In contrast, Chen et al.^20^ noted that age had no significant effect on the complete resolution of ONP after endovascular and surgical treatment. Similarly, Gu et al.^15^ and Yanaka et al.^21^ showed that age and sex were not indicative of the recovery of ONP. In our study, we found that gender and age were not significantly associated with the degree of postoperative resolution of ONP (*p* = 0.52, *p* = 0.11, respectively).

### Correlation of clinical data with oculomotor nerve palsy recovery

Stiebel-Kalish et al.^18^ observed that patients with microvascular risk factors had lesser chances of nearly complete recovery, compared to non-vascular risk factor groups. However, another study did not find any correlation between vascular risk factors and ONP recovery.^22^ In our study, we observed that co-morbidities did not play a key role in oculomotor nerve recovery (*p* = 0.64), and, most likely, recovery was dependent on other factors.

Previous researches have studied the effect of the pre-operative degree of oculomotor nerve paresis in determining eventual recovery. Many studies noted that the degree of ONP before treatment was an important factor in determining the eventual recovery of oculomotor nerve function.^7,8,23,24^ On the contrary, Guresir et al.^25^ and Patel et al.^26^ showed that the degree of ONP at presentation was not indicative of neural function recovery. In our study, amongst the 32 patients who presented initially with complete ONP, recovery of ONP was complete in 43.75%, incomplete in 43.75% and no improvement was observed in 12.5% of patients. On the other hand, the two patients who presented with partial ONP at presentation achieved complete recovery (100%). However, the differences were not statistically significant (*p* = 0.60). This is difficult to evaluate as we had only two patients who initially presented with partial ONP at admission.

The effect of aneurysm rupture status on oculomotor nerve recovery has been debated. Some studies have noted that the aneurysm rupture status before treatment was not an important factor in determining the eventual recovery of ONP.^20,27^ In contrast, other studies have found that SAH was a statistically significant factor influencing recovery.^28,29^ In our study, we found that patients with ruptured aneurysms achieved more complete recovery of ONP, compared to unruptured aneurysms (48.1% vs. 42.9%), whilst incomplete recovery was observed more in patients with unruptured aneurysm compared to the ruptured group (57.1% vs. 37%). However, the differences were not statistically significant (*p* = 0.60). The better ophthalmologic outcome with SAH can be explained by the probable difference of the ONP mechanism after SAH, which is probably related to the irritating effect of subarachnoid blood on the nerve itself, rather than to direct mass effect.

The influence of early aneurysm treatment after the onset of oculomotor nerve dysfunction and ONP resolution has been debated. Many studies have noted that the duration of ONP before treatment was an important factor in determining the eventual recovery.^30,31,32^ However, other studies have found that the interval time between ONP and the intervention did not affect the degree of post-intervention resolution of ONP.^33,34^ In our study, we found that patients with early treatment (≤ 14 days) achieved more complete recovery of ONP, compared to patients with late treatment (60% vs. 29.4%), whilst the incomplete recovery was observed more in patients with late treatment, compared to the early treatment group (53% vs. 33%). Although, the early treatment showed a higher tendency towards better ONP recovery compared to late treatment, the difference did not reach statistical significance (*p* = 0.28). This finding could be explained by the idea of neuropraxia which has an excellent prognosis with full recovery within the first few weeks, whilst recovery may not take place or occur only partially, when the third nerve palsy is of a long duration (a few months), because of intraneural scarring with axonal death and Wallerian degeneration.

### Correlation of radiological data with oculomotor nerve palsy recovery

Various studies have found that smaller aneurysms (< 10 mm in size) showed a higher tendency towards better ONP recovery, although the difference in the outcome was not statistically significant.^16,29,35^ Conversely, other authors have noted that the size of an aneurysm had no predictive value.^5,20,34^ In the current study, we found that patients with aneurysms of diameter ≤ 6.7 mm achieved more complete recovery of their ONP compared to patients with aneurysms of diameter > 6.7 mm (57.9% vs. 33.3%), whilst incomplete recovery was observed more in patients with aneurysm diameters > 6.7 mm, compared to the smaller diameter aneurysm group (46.7% vs. 36.8%). In addition, 5.3% patients showed no improvement in the smaller diameter aneurysm group, compared to 20% in the larger diameter aneurysm group. Although the smaller diameter aneurysm group showed a higher tendency towards better ONP recovery compared to the larger diameter aneurysm group, the difference was not statistically significant (*p* = 0.26).

Although it seems intuitive that large PcomA aneurysms are more likely to generate mass effect and result in ONP and would influence the degree of ONP recovery, this is not supported by the literature. Severe neurovascular compression can be seen even with small aneurysms because of nerve ischaemia. Interestingly, Anan et al.^36^ found that a short distance between the ICA and the anterior-posterior clinoid process, may be associated with ONP related to a PcomA aneurysm, and even relatively small unruptured PcomA aneurysms can cause third nerve palsy if the ICA runs close to the skull base of the third nerve.

Hamer et al.^37^ have pointed out that the posterolateral-inferior direction of the aneurysmal fundus was the most frequently encountered in the presence of an oculomotor deficit. However, Giombini et al.^24^ observed that there is no significant relationship between the radiological measurement, morphology or direction of ICA-PcomA aneurysms and the clinical picture. The relationship between aneurysm direction and ONP recovery has not been studied well.^24^ Conversely, Soni et al.^2^ believed that the shape and direction of the aneurysm sac could influence the recovery of the ONP. In this study, we found that a posterolateral aneurysm direction was most frequently encountered in the presence of oculomotor deficit (55.9%). Patients with non-posterolateral aneurysm directions achieved more complete recovery of their ONP, compared to patients with posterolateral aneurysm directions (66.7% vs. 31.6%), whilst incomplete recovery was observed more in the posterolateral group, compared to the non-posterolateral group (47.4% vs. 33.3%). Furthermore, 21% of the posterolateral group had no improvement, suggesting that the non-posterolateral aneurysm direction has a higher tendency towards better ONP recovery, compared to posterolateral aneurysm direction (*p* = 0.06). This is an interesting finding, which might be because of the anatomical relationship between the aneurysm sac and the compression on the third nerve. With a posterolateral aneurysm direction, more compression of the nerve can be expected, which could lead to increased axonal damage and poor recovery later on. On the other hand, the non-posterolateral aneurysm direction might have less pressure effect on the third nerve and a subsequently better possibility of recovery.

## Limitations

The main limitations of our study are the retrospective nature of the research and the relatively small sample size, which might influence the statistical significance.

## Conclusion

This is the first study in South Africa that examined the oculomotor recovery related to PcomA aneurysms after endovascular management. We found that the recovery of ONP after coiling of PcomA aneurysms occurred in 88.3% of the patients. Non-posterolateral aneurysm direction showed a higher tendency towards better recovery (*p* = 0.06). Smaller aneurysm size and shorter length of time of ONP before treatment, showed better recovery, but these factors were not of statistical significance. Other possible factors, such as patient gender, co-morbidities and pre-operative degree of ONP, had no predictive value in our patient group. For aneurysms amenable to both endovascular coiling and surgical clipping, including PcomA aneurysms with ONP, we recommend endovascular management as the first option.
